# All-cause, premature, and cardiovascular death attributable to socioeconomic and ethnic disparities among New Zealanders with type 1 diabetes 1994–2019: a multi-linked population-based cohort study

**DOI:** 10.1186/s12889-023-17326-8

**Published:** 2024-01-25

**Authors:** Dahai Yu, Yamei Cai, Uchechukwu Levi Osuagwu, Karen Pickering, John Baker, Richard Cutfield, Brandon J. Orr-Walker, Gerhard Sundborn, Zheng Wang, Zhanzheng Zhao, David Simmons

**Affiliations:** 1 Department of Nephrology, the First Affiliated Hospital Zhengzhou University, Zhengzhou, 450052 China; 2 Primary Care Centre Versus Arthritis, School of Medicine, Keele University, Keele, ST5 5BG UK; 3 School of Medicine, Western Sydney University, Locked Bag 1797, Campbelltown, NSW 2751 Australia; 4 Diabetes Foundation Aotearoa, Otara, New Zealand; 5 Department of Diabetes and Endocrinology, Counties Manukau Health, Auckland, New Zealand; 6 Department of Diabetes and Endocrinology, Waitemata District Health Board, Auckland, New Zealand; 7 Section of Pacific Health, the University of Auckland, Auckland, New Zealand

**Keywords:** Ethnic disparity, Socioeconomic disparity, Population attributable risk, Standardised mortality ratio, Mortality, New Zealand, Type 1 diabetes

## Abstract

**Background:**

New Zealand (NZ) research into type 1 diabetes mellitus (T1DM) mortality can inform policy and future research. In this study we aimed to quantify the magnitude to which ethnicity and socioeconomic disparities influenced mortality at the population level among people with Type 1 diabetes (T1DM) in Auckland, New Zealand (NZ).

**Methods:**

The cohort data were derived from the primary care diabetes audit program the Diabetes Care Support Service (DCSS), and linked with national primary care, pharmaceutical claims, hospitalisation, and death registration databases. People with T1DM enrolled in DCSS between 1994–2018 were included. All-cause, premature, and cardiovascular mortalities were estimated by Poisson regression models with adjustment for population-level confounders. The mortality rates ratio (MRR) was standardized against the DCSS type 2 diabetes population. Mortality rates were compared by ethnic group (NZ European (NZE) and non-NZE) and socioeconomic deprivation quintile. The population attributable fraction (PAF) was estimated for ethnic and socioeconomic disparities by Cox regression adjusting for demographic, lifestyle, and clinical covariates. The adjusted slope index inequality (SII) and relative index of inequality (RII) were used to measure the socioeconomic disparity in mortalities.

**Results:**

Overall, 2395 people with T1DM (median age 34.6 years; 45% female; 69% NZE) were enrolled, among whom the all-cause, premature and CVD mortalities were 6.69 (95% confidence interval: 5.93–7.53), 3.30 (2.77–3.90) and 1.77 (1.39–2.23) per 1,000 person-years over 25 years. The overall MRR was 0.39 (0.34–0.45), 0.65 (0.52–0.80), and 0.31 (0.24–0.41) for all-cause, premature and CVD mortality, respectively. PAF attributable to ethnicity disparity was not significantly different for mortality. The adjusted PAF indicated that 25.74 (0.84–44.39)% of all-cause mortality, 25.88 (0.69–44.69)% of premature mortality, 55.89 (1.20–80.31)% of CVD mortality could be attributed to socioeconomic inequality. The SII was 8.04 (6.30–9.78), 4.81 (3.60–6.02), 2.70 (1.82–3.59) per 1,000 person-years and RII was 2.20 (1.94–2.46), 2.46 (2.09–2.82), and 2.53 (2.03–3.03) for all-cause, premature and CVD mortality, respectively.

**Conclusions:**

Our results suggest that socioeconomic disparities were responsible for a substantial proportion of all-cause, premature and CVD mortality in people with T1DM in Auckland, NZ. Reducing socioeconomic barriers to management and self-management would likely improve clinical outcomes.

**Supplementary Information:**

The online version contains supplementary material available at 10.1186/s12889-023-17326-8.

## Background

Mortality rates among those with type 1 diabetes mellitus (T1DM) diagnosed in childhood and adolescence have been decreasing since the 1970’s, albeit with marked international variation [1]. Despite this trend, T1DM is still associated with an increased risk of death compared with the age- and sex- matched general population [2]. Premature death and deaths with cardiovascular disease (CVD) as the primary cause remain higher among people with T1DM [3–6]. The importance of minimising hyperglycaemia was confirmed by the Diabetes Control and Complications Trial (DCCT), while the Epidemiology of Diabetes Interventions and Complications (EDIC) follow up study showed a reduction in all-cause mortality and major CVD events [7].

Beyond the DCCT/EDIC (n = 1441) [7], the Allegheny County type 1 diabetes registry (n = 1075) [8] and the Finnish Diabetic Nephropathy Study (n = 5396) [9], recent large T1DM cohort mortality studies are limited. In 2019, the International Diabetes Federation calculated all-cause mortality rates but for many countries, these required substantial extrapolation [10]. In 2014, Morgan et al. compared 23 studies of T1DM mortality across 20 countries [1] revealed that after a follow up of up to 19 years, those diagnosed under 15–19 years had mortality rates of 0–8.12/1000 person years and standardised mortality rates of between 0 and 854 (100 is the background rate). A 1984–1993 New Zealand (NZ) cohort study of those with insulin treated diabetes in Canterbury showed higher all-cause mortality for those with T1DM compared to those people with type 2 diabetes (T2DM) [11]. There are no cohorts of people with T1DM in NZ reporting population-level rates of all-cause, premature and CVD mortality, with a duration of 25 years.

One of the key questions beyond the degree of excess morbidity and mortality compared with the general population [2, 12], is how these compare with those with T2DM. Generally, T1DM has been seen as more “severe” than T2DM because of the dependency on insulin treatment for survival [13]. However, the Diabetes Incidence in Kronoberg Study revealed a standardized mortality ratio of 0.5 in the Swedish T1DM population compared with the T2DM population [14]. Other studies comparing T1DM and T2DM mortality rates have suggested that reduced access to modern care and medications are likely to affect this comparison [13]. Further influences have been identified including low socio-economic status and being from a minority ethnic group, which have been associated with higher mortality [15]. Reduction of these disparities in mortality is a key health policy goal of all governments [16]. However, efforts to lower mortality rates among people with T1DM, especially premature mortality rates and to mitigate these disparities are impeded by a paucity of information on the relative contributions of ethnicity and social circumstances. New Zealand is a developed country with a multi-ethnic population that can provide insights into this issue.

In the current study, using T1DM audit data linked with national databases, we aimed to 1) investigate the all-cause, premature and CVD mortality rates in people with T1DM between 1994–2019 in NZ; 2) estimate the standard mortality ratio with the T2DM population as a reference; 3) quantify socioeconomic and ethnic disparities in mortality rates in NZ.

## Methods

### Data setting

In this open patient cohort study, we extracted data from the Diabetes Care Support Service (DCSS) dataset, a service established in 1991 to audit general practice diabetes management in South, East and West Auckland to improve standards of care [17]. The DCSS database was linked with data from national death registration, hospitalisation, pharmaceutical claim, and socioeconomic status (SES) databases. The North Health Ethics Committee approved the DCSS for research purposes in 1992, and then as an ongoing audit in 1996 (92/006). Approval for waiver of individual informed consent and approval for study was provided by the New Zealand Health Disability Ethics Committee on March 25, 2019. Anonymised data were used for this analysis and all methods were carried out in accordance with relevant guidelines and regulations.

### Participants

In this cohort study, we included all people aged ≥ 18 years with T1DM in Auckland, NZ, from the linked, de-identified DCSS database, who enrolled in DCSS between 1994–2018. T1DM was defined by primary care record coding, with validation by trained diabetes audit nurses. To minimise misclassification bias, 326 people without insulin treatment were excluded. Further validation was possible by linking hospitalisation and other registration (any database with ICD codes) data where these occurred. This involved confirming that the DCSS cases also had ICD coding for T1DM recorded: Every person remaining in the cohort had at least one T1DM record within the linked datasets.

### Exposure

NZDep2013 was used to define socioeconomic status. NZDep2013 score was derived from each New Zealand meshblock. Meshblocks are geographical units defined by Statistics New Zealand, containing a median of approximately 81 people in 2013 [18]. The original NZDep2013 scale of deprivation ranges from 1 to 10 and divides New Zealand into tenths of the distribution of the first principal component scores. To maintain statistical power, the index was re-ranked into quintiles (the most affluent group [quintile 1] to the most deprived group [quintile 5]. Ethnicity was defined by self-identity according to level 2 ethnicity codes from Ministry of Health, and cross-validated within linked databases. To maintain statistical power, ethnicity was collapsed into two groups: NZE and non-NZE (199 Māori, 158 Pasifika, 92 Asian, and 290 other ethnic groups).

### Outcomes

We collected and assessed data on three death events (all-cause death, premature death and cardiovascular death) identified between 1 January 1994 and 31 December 2019. Date and cause of death were ascertained from the linked national death registration database, which includes all deaths in New Zealand. Premature mortality was defined as all-cause death before 65 years of age. Cardiovascular death was defined by the primary International Classification of Disease (ICD) -9 (410, 411, 412, 413, 414; 430–438) and ICD-10 (I20-I25, I60-I69, I73). The count of individual deaths was used to estimate mortality.

### Covariates

Demographics data (age and sex), diabetes clinical data (including smoking, diabetes duration, body mass index (BMI), systolic blood pressure (SBP), diastolic blood pressure (DBP), HbA1c, total cholesterol (TC), triglyceride, low-density lipoprotein cholesterol (LDL-C), high-density lipoprotein cholesterol (HDL-C)) and diabetes medications (antihypertensive, anti-diabetes, statin, antiplatelet and/or anticoagulant treatment) were derived from DCSS. These data have been validated through enumeration assessment and internal quality control policies with auditors regularly cross checking, random and routine sampling/checking of data entry, and active data management (e.g., queries, checking unusual numbers, ranking of columns, duplicate checking) [17, 19, 20]. Pharmaceutical claims data includes all prescriptions issued for people and was used to cross-validate the prescription data in DCSS. Only pharmaceutical claims data after 2006 were available for data linkage. Historical claims before 2006 were not linked because National Health Index numbers were not universal until 2006. Data for all people from their first DCSS enrolment date (last enrolment 31/7/2018) were included. This manuscript reporting study findings was written in adherence with the Strengthening the Reporting of Observational Studies in Epidemiology (STROBE) reporting guideline.

### Statistical analysis

Descriptive statistics are presented as numbers and proportions for dichotomous variables and median (inter-quartile range (IQR)) for continuous variables. Clinical event rates of the three outcomes with their 95% confidence intervals (CIs) are shown for the whole cohort and stratified by ethnic group (NZE, and non-NZE), age group (≤ 35 years and > 35 years), sex, NZDep2013 quintile levels, duration of having diabetes (< 8 years and ≥ 8 years), and for two time periods (< 2003 and ≥ 2003).

Age-sex-standardised mortality rates ratios (MMRs) were estimated by overall, sex and age-stratification to compare mortality in the DCSS T1DM population to that of DCSS T2DM population, with 95% CIs determined by Poisson regression (Supplemental Technical Note).

Excess absolute risks for ethnicity (NZE as reference) and socioeconomic deprivation (least deprived group as reference) were estimated by overall, age by enrolment, sex, duration of having diabetes, smoking status, body mass index (BMI), blood pressure, HbA1c, lipids, estimated Glomerular filtration rate (eGFR), antihypertensive medicine, and statin. We used the data for each outcome for people in the least deprived group or from a NZE ethnic background as the reference rate, which were then applied to the entire population to estimate the expected numbers of people with each of the three outcomes. Attributable fraction was defined as the difference in the observed and expected number of people with an adverse clinical outcome, divided by the observed number. The attributable fraction described the proportion of adverse clinical outcome that would not have occurred were the rates of the outcome the same as in the reference group. The attributable fraction compares the reference group with the entire population, producing a population attributable fraction [21, 22].

Cox regression models were used to estimate expected numbers with adverse outcomes, adjusting for ethnicity or deprivation, age by enrolment, sex, duration of having diabetes, duration of having diabetes, smoking status, body mass index (BMI), blood pressure, HbA1c, lipids, estimated Glomerular filtration rate (eGFR), antihypertensive medicine, and statin. Missing data on the covariates ranged 0–6% of eligible participants with T1DM, based on the worst-case scenario of 6% of cohort members with one or more missing covariates, we created 6 imputed datasets using the multiple imputation with chained equations [23]. Final adjusted estimations were derived from imputed models.

The population-weighted, regression-based slope index of inequality (SII) and relative index of inequality (RII) was estimated for socioeconomic inequality in each outcome, which are interpreted as the effect on the health of moving from the least to the most deprived group (Supplemental Table 1) using the adjusted outcome rates by socioeconomic status estimated from the above adjusted Poisson regression models [24]. SII is at the value zero when there is no inequality. Greater values indicate higher levels of inequality. Positive values indicate a higher concentration of a condition among the most deprived group and negative values indicate a higher concentration among the least deprived. RII is at the value one when there is no inequality. Further values from one indicate higher levels of inequality. Values larger than one indicate a concentration of a condition among the most deprived group and values smaller than one indicate a concentration among the least deprived. SII and RII were estimated by overall, age by enrolment, sex, duration of having diabetes, duration of having diabetes, smoking status, body mass index (BMI), blood pressure, HbA1c, lipids, estimated Glomerular filtration rate (eGFR), antihypertensive medicine, and statin. The confidence interval for each SII and RII were estimated by bootstrapping method with resampling 10,000 times.

All statistical analyses were done by use of Stata MP 17.0 taking a 2 tailed *P*-value < 0.05 as significant.

## Results

### Baseline characteristics

As shown as Table 1, 2,395 people with T1DM were enrolled in the DCSS between Jan 1, 1994, and July 31, 2018; 1,080 (45.1%) were females and the median age was 34.6 (IQR: 21.1 to 47.9) years. Follow up occurred for a median of 10.1 years (IQR 6.2–14.0). The median duration of having diabetes was 8 (2 to 18) years. Similar proportions (21.3%) were in the least and the most deprived group. The clinical measurements and proportion taking antihypertensive medicine and statin are shown in Table 1. Table 1 also shows the characteristics of the 1,656 (69.1%) NZE, and non-NZE 739 (30.9%). Metabolic characteristics were similar between NZE and non-NZE populations. The comparison between our sample and the national registry data [25] suggests our sample exhibits key demographic similarities to the national data, supporting its representativeness for the studied population (Supplemental Table 2).”

**Table 1:** Characteristics of study participants at enrolment into the diabetes care support service

	**All**	**NZE**	**Non-NZE**
N	2395	1656	739
Age at enrolment. years	34.6 (21.1 to 47.9)	35.1 (21.6 to 47.9)	33.3 (19.6 to 48.0)
Age stratification			
≤ 35 years	1218 (50.9)	825 (49.8)	393 (53.2)
> 35 years	1177 (49.1)	831 (50.2)	346 (46.8)
Sex			
Female	1080 (45.1)	715 (43.2)	365 (49.4)
Male	1315 (54.9)	941 (56.8)	374 (50.6)
NZDep13 score quintile			
Quintile-1	510 (21.3)	403 (24.4)	107 (14.4)
Quintile-2	464 (19.4)	359 (21.7)	105 (14.2)
Quintile-3	412 (17.2)	303 (18.3)	109 (14.7)
Quintile-4	499 (20.8)	318 (19.2)	181 (24.4)
Quintile-5	510 (21.3)	271 (16.4)	239 (32.3)
Current Smoker, n (%)	308 (12.9)	183 (11.1)	125 (16.9)
Duration of having diabetes, years	8 (2 to 18)	9 (2 to 19)	6 (1 to 15)
Body mass index, kg/m^2^	25.0 (22.3 to 28.6)	25.0 (22.1 to 28.1)	25.6 (22.8 to 30.0)
Obesity, n (%)	456 (19.0)	273 (16.5)	192 (26.0)
Systolic blood pressure, mmHg	120 (110 to 135)	120 (110 to 135)	120 (110 to 137)
Diastolic blood pressure, mmHg	76 (70 to 80)	76 (70 to 80)	77 (70 to 80)
HbA1c, mmol/mol (SD) / %	67.2 (56.3 to 80.3) / 8.3 (7.3 to 9.5)	66.1 (55.2 to 77.1) / 8.2 (7.2 to 9.2)	70.7 (58.6 to 87.0) / 8.6 (7.5 to 10.1)
Total cholesterol, mmol/L	4.9 (4.3 to 5.7)	4.9 (4.3 to 5.6)	5.0 (4.2 to 5.8)
Triglyceride, mmol/L	1.1 (0.8 to 1.7)	1.1 (0.8 to 1.6)	1.3 (0.8 to 2.2)
Low density lipoprotein, mmol/L	2.5 (2.0 to 3.0)	2.5 (2.0 to 3.0)	2.5 (2.0 to 3.2)
High density lipoprotein, mmol/L	1.4 (1.2 to 1.7)	1.4 (1.2 to 1.8)	1.3 (1.1 to 1.6)
estimated Glomerular filtration rate (eGFR) < 90 ml/min/1.73 m^2^, n (%)	983 (41.0)	719 (43.4)	264 (35.7)
Statin treatment on entry, n (%)	663 (27.7)	488 (29.5)	175 (23.7)
Taking antihypertensive treatment, n (%)	857 (35.8)	630 (38.0)	227 (30.7)

Continuous variables were presented as median (inter-quartile range); binary and categorical variables were presented as number (percentage)

95% Confidence Interval shown

### Population-level outcomes

The overall rate of all-cause mortality (/1000 person years) was 6.69 (95% confidence interval: 5.93–7.53). Higher rates were observed among those aged more than 35 years, the most socioeconomically deprived, and those enrolled before 2003 (Table 2).

**Table 2:** Mortality rates of clinical events among people with type 1 diabetes in DCSS between 1994–2018

	**All-cause mortality**	**Premature mortality**	**CVD mortality**
Overall numerators, n	276	136	73
Overall denominators, person-years	41,244.853	41,244.853	41,244.853
All	6.69 (5.93 to 7.53)	3.30 (2.77 to 3.90)	1.77 (1.39 to 2.23)
Gender			
Male	6.61 (5.59 to 7.76)	3.59 (2.85 to 4.47)	1.78 (1.27 to 2.42)
Female	6.79 (5.66 to 8.07)	2.94 (2.21 to 3.83)	1.76 (1.21 to 2.48)
Age stratification			
≤ 35 years	2.18 (1.59 to 2.91)	2.18 (1.59 to 2.81)	0.47 (0.23 to 0.87)
> 35 years	11.43 (10.00 to 13.00)	4.47 (3.60 to 5.50)	3.13 (2.41 to 4.00)
NZDep13 score quintile			
Quintile-1	3.64 (2.49 to 5.14)	1.48 (0.79 to 2.53)	0.80 (0.32 to 1.64)
Quintile-2	5.41 (3.93 to 7.26)	2.83 (1.79 to 4.24)	1.35 (0.68 to 2.42)
Quintile-3	6.58 (4.87 to 8.70)	2.96 (1.85 to 4.48)	1.61 (0.83 to 2.82)
Quintile-4	7.40 (5.70 to 9.45)	3.35 (2.25 to 4.82)	1.97 (1.15 to 3.15)
Quintile-5	10.57 (8.47 to 13.04)	5.96 (4.41 to 7.87)	3.16 (2.06 to 4.63)
Ethnicity			
NZE	6.94 (6.01 to 7.98)	3.11 (2.49 to 3.82)	1.85 (1.39 to 2.42)
Māori	8.05 (5.16 to 11.98)	6.38 (3.84 to 9.96)	1.68 (0.54 to 3.92)
Pacific Islanders	8.29 (5.06 to 12.80)	4.97 (2.57 to 8.68)	1.24 (0.26 to 3.63)
Other ethnic group	4.59 (3.16 to 6.44)	2.22 (1.27 to 3.61)	1.67 (0.86 to 2.91)
Smoking status			
Non-/ex-smoker	6.65 (5.84 to 7.55)	3.05 (2.51 to 3.67)	1.75 (0.80 to 3.31)
Current smoker	6.98 (4.89 to 9.67)	5.04 (3.29 to 7.39)	1.77 (1.37 to 2.26)
Enrolment period			
< 2003	7.67 (6.69 to 8.76)	3.57 (2.91 to 4.34)	2.24 (1.73 to 2.86)
≥ 2003	4.49 (3.40 to 5.81)	2.68 (1.85 to 3.74)	0.71 (0.32 to 1.34)
Duration of having diabetes			
< 8 years	3.98 (3.13 to 5.00)	2.91 (2.18 to 3.79)	1.02 (0.62 to 1.60)
≥ 8 years	8.91 (7.72 to 10.23)	3.62 (2.88 to 4.49)	2.38 (1.79 to 3.11)

The rate was presented as per 1,000 person-years

The overall rates of premature and CVD mortality (/1000 person years) were 3.30 (95% confidence interval: 2.77–3.90) and 1.77 (1.39–2.23) (/1000 person years) respectively, over the study period. Higher premature and CVD mortality rates were observed among those aged more than 35 years and the most socioeconomically deprived (Table 2). Higher all-cause and premature mortality rates were found in Māori and Pacific Islander ethnic groups, while a higher CVD mortality rate was observed in the NZE ethnic group (Table 2).

Excess risk (attributable risk) of all-cause, premature, and CVD mortality for ethnicity (with NZE as reference) was not significantly different either overall ( -0.83 (-2.50 to 0.85), 0.63 (-0.62 to 1.88) and -0.26 (-1.12 to 0.60) per 1,000 person-years, respectively) or by gender, age-group, duration of diabetes, smoking status, enrolment period, obesity status, HbA1c, SBP, TC, and eGFR (Supplemental Table 3).

Excess risk (attributable risk) of all-cause, premature and CVD mortality for socioeconomic deprivation (with least the deprived group as reference) increased with deprivation level and peaked at the most deprived group overall. The same pattern was found for all-cause and premature mortality among males and among females separately (but not significant for CVD mortality) (Supplemental Table 3), by age-group, duration of diabetes, smoking status, enrolment period, obesity status, HbA1c, SBP, TC, and eGFR.

The adjusted mortality rate ratios and adjusted excess mortality rates by ethnicity and deprivation have been presented in Supplemental Table 4. When compared with the NZE group, the adjusted mortality rate ratios and adjusted excess mortality rates did not show statistical significance for each death outcome. However, when comparing with the least deprived group, we found significantly higher mortality rate ratios and adjusted excess mortality rates in the more deprived group. The highest measurements were observed in the most deprived group for each death outcome.

The overall adjusted absolute inequality (SII) for all-cause, premature and CVD mortality was 8.04 (6.30 to 9.78), 4.81 (3.60 to 6.02) and 2.70 (1.82 to 3.59) per 1,000 person-years respectively; RII was 2.20 (1.94 to 2.46), 2.46 (2.09 to 2.82), 2.53 (2.03 to 3.03) respectively as shown in Table 3. No ethnic differences were found. Higher SII was more likely to be found in those aged over 35 years (vs ≤ 35 years), obesity group (all-cause mortality only), people with higher SBP (all-cause and premature mortality), TC (all-cause and CVD mortality) and eGFR (CVD mortality only), (Table 3). RII was lower among those with eGFR ≥ 90 ml/min/1.73 m^2^ for all-cause and premature mortality.

**Table 3:** Slope index of inequality and relative index of inequality for mortality by overall, sex, age-group, and clinical measurements

	**All-cause mortality**		**Premature mortality**		**CVD mortality**	
	**SII (95% CI), per 1,000 person-years**	**RII**	**SII (95% CI), per 1,000 person-years**	**RII**	**SII (95% CI), per 1,000 person-years**	**RII**
**Overall**^**a**^	8.04 (6.30 to 9.78)	2.20 (1.94 to 2.46)	4.81 (3.60 to 6.02)	2.46 (2.09 to 2.82)	2.70 (1.82 to 3.59)	2.53 (2.03 to 3.03)
**Ethnicity**^**b**^						
**NZE**	9.42 (7.22 to 11.62)	2.30 (2.00 to 2.60)	4.54 (3.07 to 6.01)	2.40 (1.95 to 2.85)	3.33 (2.21 to 4.46)	2.71 (2.13 to 3.29)
**Non-NZE**	7.18 (4.32 to 10.05)	2.18 (1.77 to 2.79)	5.77 (3.62 to 7.93)	2.73 (2.08 to 3.37)	2.13 (0.72 to 3.54)	2.45 (1.49 to 3.41)
**Sex**^**c**^						
**Men**	8.19 (5.83 to 10.55)	2.22 (1.87 to 2.57)	5.36 (3.63 to 7.09)	2.47 (1.99 to 2.94)	2.83 (1.62 to 4.04)	2.57 (1.90 to 3.24)
**Women**	7.86 (5.28 to 10.43)	2.17 (1.79 to 2.56)	4.31 (2.64 to 5.98)	2.49 (1.91 to 3.07)	2.54 (1.24 to 3.85)	2.46 (1.71 to 3.20)
**Age-group**^**d**^						
≤ **35 years**	2.79 (1.39 to 4.19)	2.25 (1.62 to 2.87)	2.79 (1.39 to 4.20)	2.25 (1.62 to 2.88)	0.98 (0.35 to 1.62)	2.98 (1.70 to 4.27)
> **35 years**	12.97 (9.73 to 16.21)	2.14 (1.86 to 2.43)	6.79 (4.80 to 8.78)	2.53 (2.08 to 2.98)	4.35 (2.67 to 6.03)	2.40 (1.86 to 2.94)
**Smoking status**^**e**^						
**Non-smoker/ex-smoker**	8.59 (6.72 to 10.46)	2.27 (2.00 to 2.55)	4.60 (3.34 to 5.86)	2.48 (2.08 to 2.89)	2.81 (1.86 to 3.77)	2.56 (2.03 to 3.09)
**Current Smoker**	4.28 (-0.76 to 9.32)	1.64 (0.89 to 2.38)	4.82 (0.65 to 9.00)	2.02 (1.14 to 2.90)	2.10 (-0.32 to 4.53)	2.30 (0.81 to 3.79)
**Enrolment period**^**f**^						
≤ **2003**	8.17 (5.93 to 10.42)	2.07 (1.78 to 2.36)	4.17 (2.65 to 5.69)	2.17 (1.74 to 2.60)	3.28 (2.08 to 4.47)	2.47 (1.93 to 3.00)
> **2003**	7.79 (5.24 to 10.34)	2.72 (2.16 to 3.29)	6.15 (4.25 to 8.04)	3.30 (2.59 to 4.01)	1.21 (0.13 to 2.30)	2.44 (1.15 to 3.73)
**Duration of having diabetes**^**g**^						
< **8 years**	6.99 (5.01 to 8.97)	2.74 (2.24 to 3.23)	5.36 (3.66 to 7.05)	2.80 (2.23 to 3.37)	2.45 (1.49 to 3.41)	3.40 (2.46 to 4.34)
≥ **8 years**	8.85 (6.14 to 11.56)	2.00 (1.69 to 2.30)	4.36 (2.65 to 6.06)	2.22 (1.74 to 2.69)	2.90 (1.50 to 4.29)	2.22 (1.63 to 2.80)
**Obesity status**^**h**^						
< **25 kg/m**^**2**^	4.67 (2.04 to 7.30)	1.87 (1.38 to 2.37)	3.21 (1.19 to 5.23)	1.99 (1.37 to 2.62)	2.62 (1.36 to 3.88)	2.96 (2.02 to 3.91)
≥ **25 kg/m**^**2**^	9.59 (7.33 to 11.85)	2.29 (1.99 to 2.60)	5.63 (4.16 to 7.09)	2.70 (2.25 to 3.14)	2.65 (1.46 to 3.83)	2.30 (1.72 to 2.88)
**HbA1c level**^**i**^						
< **70 mmol/mol**	8.39 (6.14 to 10.64)	2.51 (2.11 to 2.92)	3.87 (2.55 to 5.19)	2.87 (2.24 to 3.51)	2.69 (1.54 to 3.84)	2.81 (2.04 to 3.58)
≥ **70 mmol/mol**	6.61 (3.92 to 9.31)	1.83 (1.49 to 2.18)	4.80 (2.78 to 6.82)	2.07 (1.62 to 2.51)	2.59 (1.25 to 3.93)	2.27 (1.61 to 2.92)
**Systolic blood pressure level**^j^						
< **120 mmHg**	3.09 (1.40 to 4.79)	2.17 (1.53 to 2.81)	2.59 (1.08 to 4.10)	2.23 (1.51 to 2.95)	1.86 (0.95 to 2.77)	3.60 (2.32 to 4.88)
≥ **120 mmHg**	10.95 (8.28 to 13.62)	2.17 (1.88 to 2.45)	6.17 (4.44 to 7.89)	2.51 (2.09 to 2.93)	3.16 (1.80 to 4.53)	2.28 (1.73 to 2.83)
**Total cholesterol level**^**k**^						
< **5.0 mmol/L**	4.21 (2.41 to 6.01)	1.98 (1.56 to 2.40)	3.31 (2.09 to 4.53)	2.63 (2.03 to 3.23)	1.03 (0.12 to 1.95)	1.93 (1.10 to 2.75)
≥ **5.0 mmol/L**	13.07 (9.64 to 16.50)	2.27 (1.93 to 2.60)	6.62 (4.19 to 9.05)	2.26 (1.80 to 2.72)	5.07 (3.35 to 6.79)	2.85 (2.22 to 3.48)
**Estimated glomerular filtration rate level**^**l**^						
< **90 ml/min/1.73 m**^**2**^	6.38 (4.75 to 8.00)	2.82 (2.36 to 3.29)	5.30 (3.94 to 6.66)	3.10 (2.56 to 3.64)	1.44 (0.76 to 2.11)	3.17 (2.15 to 4.20)
≥ **90 ml/min/1.73 m**^**2**^	11.35 (7.92 to 14.79)	2.03 (1.72 to 2.34)	4.44 (2.30 to 6.58)	2.03 (1.53 to 2.53)	4.78 (2.92 to 6.64)	2.45 (1.89 to 3.02)

SII and RII indicates slope index of inequality and relative index of inequality, respectively

^a^Ethnicity, age, sex, duration of having diabetes, enrolment year, smoking status, BMI, SBP, DBP, HbA1c, TC, triglyceride, LDL-C, HDL-C, eGFR, statin and antihypertensive medicine were adjusted

^b^Age, sex, duration of having diabetes, enrolment year, smoking status, BMI, SBP, DBP, HbA1c, TC, triglyceride, LDL-C, HDL-C, eGFR, statin and antihypertensive medicine were adjusted

^c^Ethnicity, age, duration of having diabetes, enrolment year, smoking status, BMI, SBP, DBP, HbA1c, TC, triglyceride, LDL-C, HDL-C, eGFR, statin and antihypertensive medicine were adjusted

^d^Ethnicity, sex, duration of having diabetes, enrolment year, smoking status, BMI, SBP, DBP, HbA1c, TC, triglyceride, LDL-C, HDL-C, eGFR, statin and antihypertensive medicine were adjusted

^e^Ethnicity, age, sex, duration of having diabetes, enrolment year, BMI, SBP, DBP, HbA1c, TC, triglyceride, LDL-C, HDL-C, eGFR, statin and antihypertensive medicine were adjusted

^f^ Ethnicity, age, sex, duration of having diabetes, smoking status, BMI, SBP, DBP, HbA1c, TC, triglyceride, LDL-C, HDL-C, eGFR, statin and antihypertensive medicine were adjusted

^g^Ethnicity, age, sex, enrolment year, smoking status, BMI, SBP, DBP, HbA1c, TC, triglyceride, LDL-C, HDL-C, eGFR, statin and antihypertensive medicine were adjusted

^h^Ethnicity, age, sex, duration of having diabetes, enrolment year, smoking status, blood pressure, HbA1c, TC, triglyceride, LDL-C, HDL-C, eGFR, statin and antihypertensive medicine were adjusted

^i^Ethnicity, age, sex, duration of having diabetes, enrolment year, smoking status, BMI, SBP, DBP, TC, triglyceride, LDL-C, HDL-C, eGFR, statin and antihypertensive medicine were adjusted

^j^Ethnicity, age, sex, duration of having diabetes, enrolment year, smoking status, BMI, SBP, DBP, HbA1c, TC, triglyceride, LDL-C, HDL-C, eGFR, statin and antihypertensive medicine were adjusted

^k^Ethnicity, age, sex, duration of having diabetes, enrolment year, smoking status, BMI, SBP, DBP, HbA1c, TC, triglyceride, LDL-C, HDL-C, eGFR, statin and antihypertensive medicine were adjusted

Ethnicity, age, sex, duration of having diabetes, enrolment year, smoking status, BMI, SBP, DBP, HbA1c, eGFR, statin and antihypertensive medicine were adjusted

^l^Ethnicity, age, sex, duration of having diabetes, enrolment year, smoking status, BMI, SBP, DBP, HbA1c, TC, triglyceride, LDL-C, HDL-C, statin and antihypertensive medicine were adjusted

The population attributable fraction for ethnicity was -4.33 (-12.39 to 3.15) %, 5.36 (-6.90 to 16.22) %, and -5.02 (-20.97 to 8.83) % unadjusted for all-cause, premature and CVD mortality respectively. Similar patterns were seen after adjustment for socioeconomic group, age, sex, duration of having diabetes, enrolment year, smoking status, BMI, SBP, DBP, HbA1c, TC, triglyceride, LDL-C, HDL-C, eGFR, statin and antihypertensive medicine (Table 4; Supplemental Table 5).

**Table 4:** Population attributable fractions of mortality restriction by socioeconomic deprivation and ethnicity

	**All-cause mortality**	**Premature mortality**	**CVD mortality**
Ethnicity^a^	5.93 (-8.47 to 18.43) %	12.03 (-11.64 to 30.68) %	-13.02 (-57.74 to 19.03) %
Socioeconomic deprivation^b^	25.74 (0.84 to 44.39) %	25.88 (0.69 to 44.69) %	55.89 (1.20 to 80.31) %

BMI stands for Body Mass Index, SBP for Systolic Blood Pressure, DBP for Diastolic Blood Pressure, TC for Total Cholesterol, LDL-C for Low-Density Lipoprotein Cholesterol, HDL-C for High-Density Lipoprotein Cholesterol, and eGFR for Estimated Glomerular Filtration Rate

^a^Socioeconomic group, age, sex, duration of having diabetes, enrolment year, smoking status, BMI, SBP, DBP, HbA1c, TC, triglyceride, LDL-C, HDL-C, eGFR, statin and antihypertensive medicine were adjusted

^b^Ethnicity, age, sex, duration of having diabetes, enrolment year, smoking status, BMI, SBP, DBP, HbA1c, TC, triglyceride, LDL-C, HDL-C, eGFR, statin and antihypertensive medicine were adjusted

The population attributable fraction for socioeconomic deprivation was 46.60 (26.11 to 61.41) %, 55.99 (26.39 to 73.68) %, and 55.82 (10.70 to 78.14) % unadjusted for all-cause, premature and CVD mortality respectively. Adjustment for ethnicity, age, sex, duration of having diabetes, enrolment year, smoking status, BMI, SBP, DBP, HbA1c, TC, triglyceride, LDL-C, HDL-C, eGFR, statin and antihypertensive medicine, had no significant effect on the association between socioeconomic deprivation and all-cause, premature and CVD mortality (Table 4; Supplemental Table 5).

**Fig. 1:**
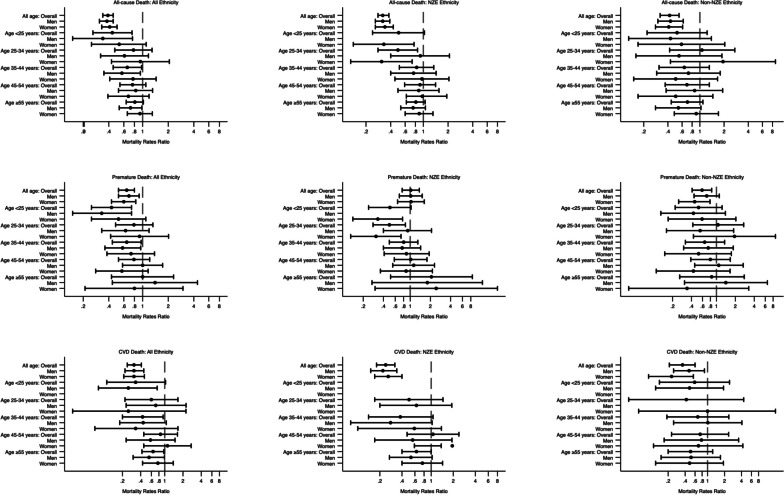
Mortality rates ratio of clinical events among people with type 1 diabetes in DCSS between 1994–2018. DCSS type 2 diabetes population was the reference group. Missing bars indicates the no recorded events for the sub-group people with type 1 diabetes

The overall all-cause mortality, premature mortality and CVD MRRs in the DCSS T1DM population were 0.39 (0.34 to 0.45), 0.65 (0.52 to 0.80), and 0.31 (0.24 to 0.41), respectively (Fig. 1; Supplemental Table 6). Age-specific, sex-specific and ethnic-specific MRRs are shown in Fig. 1 & Supplemental Table 6.

## Discussion

Consistent with the Diabetes Incidence in Kronoberg Study, the current study found that all-cause, premature and CVD mortality in a T1DM cohort in NZ was 0.39, 0.65, and 0.31 times lower than that in the T2DM population, standardising for age and sex. There was no significant excess all-cause, premature and CVD mortality among non-NZE compared with NZE, while mortality increased significantly with increments in socioeconomic deprivation. The absolute socioeconomic inequality measurement suggested that 8, 5, and 3 of annual all-cause, premature and CVD additional avoidable deaths per 1,000 with T1DM occurred in the most deprived group compared with the least deprived group.

The population attributable fraction of ethnicity for the three mortality outcomes was not significantly different including after adjustment for potential socio-demographic and clinical factors. Conversely, 47% of all-cause mortality, 56% of premature mortality, and 56% of CVD mortality would not have occurred if all people with T1DM had the same risk as those in the least deprived socioeconomic group. These population attributable fractions were lower for all-cause and premature mortality when adjusted for ethnicity, age, sex, diabetes duration, enrolment year, smoking status, BMI, SBP, DBP, HbA1c, TC, triglyceride, LDL-C, HDL-C, eGFR, statin and antihypertensive medicine, which suggests that some of the socioeconomic disparities in all-cause and premature but not CVD mortality can be explained by the combined influences of these clinical characteristics. Adjustment for these characteristics had little effect on the population attributable fraction for socioeconomic disparities in CVD mortality.

Studies comparing mortality among people with T1DM and T2DM vary between different populations and by ethnicity. In the Finnish cohort including 211 people with T1DM and an 18-year follow up, CVD mortality was similar between people with T1DM and T2DM [26]. In an Australian cohort including 470 people with T1DM aged 15–30 years over 23 years of follow up, 0.5-fold and 0.6-fold less all cause and CVD mortality was found in the T1DM population compared with the T2DM population [27]. In a Hungarian cohort including 11,863 people with T1DM with a mean age of 22 years, a twofold higher risk of all-cause mortality was identified in the T1DM group compared with the T2DM group [28]. In a Korean cohort including 9397 people with T1DM aged 20 years and over, people with T1DM had a 1.9-fold higher risk of all-cause mortality compared to people with T2DM [29]. In the current cohort with 2395 people with T1DM and a median age of 35 years, overall median follow-up of 10 years, overall, 0.39-fold, 0.65-fold, and 0.31-fold decreased risk of all-case, premature, and CVD mortality (respectively), were identified. Similar findings were observed in the non-NZE sub-cohort. In the NZE sub-cohort, similar findings were only observed for all-cause and CVD mortality, with no difference in premature mortality between T1DM and T2DM. The inconsistent findings (higher mortality in T1DM than T2DM) might be due to different health systems, worse diabetes complications than the T2DM population over time ( eg in Asia (25); and/or the younger mean age, for example in the comparison in Europe (24).

Higher raw all-cause mortality and premature mortality rates were observed in Māori and Pacific ethnic groups, while a higher raw CVD mortality rate was observed in the NZE ethnic group. Due to sample size limitations, adjusted mortality rate ratios and adjusted excess mortality were not estimated for specific comparisons between each ethnic group and NZE. Furthermore, comparisons between NZE and combined non-NZE groups did not reveal any significant differences in all-cause, premature, or CVD mortality after adjustment for socioeconomic status, demographic and clinical characteristics. This lack of significance may be attributed to heterogeneity within the non-NZE groups. Future studies with larger sample sizes within each specific ethnic group are needed to enable more precise ethnic-specific comparisons.

Female MRR in certain age groups, particularly within the 25–34 age range, exhibits significant variations compared to male estimations, especially in the Non-NZE population, where no significant estimations were observed. There may be heterogeneity effects within this age group that warrant further investigation through the subdivision into smaller age groups in future studies with a larger sample size. The current study was constrained by limited outcomes within this specific age group of the Non-NZE population. However, significant attributable risks in relation to socioeconomic status were demonstrated for all three mortalities. CVD mortality, in particular, had over half of the excess risk attributable to socioeconomic deprivation, including after adjustment for ethnicity, demographic and clinical factors. Multiple barriers to care and self-care have previously been shown in the 20^th^ Century among people with diabetes in South Auckland [30] including those relating to socioeconomic deprivation and hence this disparity is not unexpected. More recently, this has been shown across NZ for people with T1DM where it was suggested that the NZ funding model via the Government Drug purchasing agency PHARMAC is unable to allocate equal resource between devices/pharmaceuticals and diabetes support and education [27]. Our study, with the high proportion attributable to deprivation, suggests that greater efforts should be made to reduce these socioeconomic disparities in the three mortalities among people with T1DM. Potentially, 8.04, 4.81, and 2.70 per 1,000 person-years in the average all-cause, premature, and CVD mortality between the least and the most deprived socioeconomic group could be eliminated by applying a ‘place-based’ public health strategy [25], (https://www.gov.uk/government/publications/health-inequalities-place-based-approaches-to-reduce-inequalities/place-based-approaches-for-reducing-health-inequalities-main-report.) for people with T1DM and socioeconomic disadvantage in NZ. We have not calculated years of life lost, but the cohort had a mean entry age of 34.6 years suggesting that many of those dying would have bene of working age.

The study has a number of strengths including this being the longest (25 years) cohort study of a T1DM population in New Zealand to report all-cause, premature, and CVD mortality. The population basis of the cohort is a further strength with all patients derived from participating general practices. Through data linkage to large, nationally representative databases, we were able to follow up people to ascertain all incident cause-specific death. All health outcomes used in this study were based on the linkage of specific registration datasets, which provide high validity of outcomes. Accuracy of clinical recording and diagnoses in this study have been found to be valid for a range of comorbidities and we also used primary ICD codes for cause-specific outcomes, which have high precision [31]. The study limitations include heterogeneity of the non-NZE group and the degree of representativeness of the population (South and West Auckland vs NZ) and the participating general practices. Misdiagnosis between T1DM and T2DM is a well-known phenomenon in primary care [32], although we restricted the population to T1DM and with insulin treatment. Restricted by sample size, the population-level estimation in the current study could not be broken down to furthermore specific-ethnic groups in the non-NZE population.

## Conclusions

In conclusion, our findings suggested that there is a lower risk of all-cause, premature, and CVD mortality for the population with T1DM compared with those with T2DM in this cohort. The lack of significant differences in mortality among ethnic groups with T1DM in NZ may be due to heterogeneity within non-NZE groups necessitating larger samples for precise ethnic-specific comparisons. However, socioeconomic disparities were attributable for a major proportion of the three mortalities. Significant deaths could be reduced if prevention strategies including better access to care, education and continuous glucose monitoring target people with T1DM with socioeconomic disadvantage.

### Supplementary Information

**Additional file 1: Supplemental Table 1**. Technical notes for slope index of inequality and relative index of inequality. **Supplemental Table 2.** Comparison of Demographic Characteristics Between DCSS Type 1 Diabetes Population and the National Registry of Type 1 Diabetes. **Supplemental table 3**. Excess mortality for ethnicity and socioeconomic inequality by overall, sex, enrol age, and clinical measurements. **Supplemental table 4**. Adjusted Mortality rates ratio and excess mortality for ethnicity and socioeconomic inequality. **Supplemental Table 5.** Population attributable fractions of mortality restriction by socioeconomic deprivation and ethnicity. **Supplemental Table 6. **Mortality rates ratio (MRR) of clinical events among people with type 1 diabetes in DCSS between 1994-2018

## Data Availability

The datasets analysed in the current study are not publicly available because of agreements with the primary care organisations and Ministry of Health who provided the data but are available from the corresponding author on reasonable request.

## Abbreviations

T1DM: Type 1 diabetes mellitus
CVD: Cardiovascular diseases
T2DM: Type 2 diabetes mellitus
NZ: New Zealand
MRR: Mortality rates ratio
PAF: Population attributable fraction
TC: Total cholesterol
SBP: Systolic blood pressure
eGFR: Estimated glomerular filtration rate
SII: Slope index of inequality
RII: Relative index of inequality
NZE: New Zealand European
