# Supplementary material for: All-cause, premature, and cardiovascular death attributable to socioeconomic and ethnic disparities among New Zealanders with type 1 diabetes 1994–2019: a multi-linked population-based cohort study
Source: BMC Public Health. 2024 Jan 25;24:298. doi: 10.1186/s12889-023-17326-8 (PMC10811898; doi:10.1186/s12889-023-17326-8)
Supplement: Supplementary file 1 — Additional file 1: Supplemental Table 1. Technical notes for slope index of inequality and relative index of inequality. Supplemental Table 2. Comparison of Demographic Characteristics Between DCSS Type 1 Diabetes Population and the National Registry of Type 1 Diabetes. Supplemental table 3. Excess mortality for ethnicity and socioeconomic inequality by overall, sex, enrol age, and clinical measurements. Supplemental table 4. Adjusted Mortality rates ratio and excess mortality for ethnicity and socioeconomic inequality. Supplemental Table 5. Population attributable fractions of mortality restriction by socioeconomic deprivation and ethnicity. Supplemental Table 6. Mortality rates ratio (MRR) of clinical events among people with type 1 diabetes in DCSS between 1994-2018 [file 12889_2023_17326_MOESM1_ESM.docx]

**Supplemental Material**

Supplemental Technical Note for Poisson Regression

Multiple Poisson regression for rate is specified by adding the offset in the form of the natural log of the denominator t. This is given as,

Ln(y)=ln(t)+b_0_+b_1_x_1_+b_2_x_2_+...+b_p_x_p_

IRR_p_=exp(b_p_)

**Supplemental Table 1**. Technical notes for slope index of inequality and relative index of inequality

Both slope index of inequality (SII) and relative index of inequality (RII) are population-weighted and regression-based inequality measurements, which are interpreted as the effect on the health of moving from the least to the most deprived group.

| Regress the mortality on the midpoint of IMD categories, weighted by proportion in the population:  $Prevalence=\beta_{0}+\beta_{1}\left( IMD midpoint \right)+\varepsilon$  – Slope Index of Inequality (SII) = $\beta_{1}$ – Relative Index of Inequality (RII) = 1 + (SII/ adjusted marginal mortality rate in the whole population IMD decile 1-5  Where:  $\beta_{0}$ is the intercept of the regression line and the Y-axis $\beta_{1}$ is the coefficient that relates to the midpoint of the range of the distribution of IMD; $\varepsilon$ is an error term. |
| --- |

SII is at the value zero when there is no inequality. Greater values indicate higher levels of inequality. Positive values indicate a higher concentration of a condition among the most deprived group and negative values indicate a higher concentration among the least deprived. RII is at the value one when there is no inequality. Further values from one indicate higher levels of inequality. Values larger than one indicate a concentration of a condition among the most deprived group and values smaller than one indicate a concentration among the least deprived. SIIs and RIIs were calculated using a standard analytical tool provided by England Office for Health Improvement and Disparities.

Supplemental Table 2: Comparison of Demographic Characteristics Between DCSS Type 1 Diabetes Population and the National Registry of Type 1 Diabetes

|  | DCSS, 1994-2018 | National data collection, 2012-2016^a^ |
| --- | --- | --- |
| n | 2395 | 17338 |
| Age stratification |  |  |
| ≤35 years for DCSS; ≤39 yeas for national data | 1218 (50.9) | 9454 (44.5) |
| >35 years for DCSS; >39 for national data | 1177 (49.1) | 7884 (45.5) |
| Sex |  |  |
| Female | 1080 (45.1) | 8657 (49.9) |
| Male | 1315 (54.9) | 8681 (50.1) |
| NZDep13 score quintile |  |  |
| Quintile-1 | 510 (21.3) | 3061 (17.8) |
| Quintile-2 | 464 (19.4) | 3223 (18.7) |
| Quintile-3 | 412 (17.2) | 3459 (20.1) |
| Quintile-4 | 499 (20.8) | 4080 (23.7) |
| Quintile-5 | 510 (21.3) | 3420 (19.8) |
| Ethnicity |  |  |
| NZE | 1656 (69.1) | 13142 (75.8) |
| Non-NZE | 739 (30.9) | 4196 (24.2) |
| 1. Wheeler BJ, Braund R, Galland B, Mikuscheva A, Wiltshire E, Jefferies C, de Lange M: District health board of residence, ethnicity and socioeconomic status all impact publicly funded insulin pump uptake in New Zealand patients with type 1 diabetes. N Z Med J 2019, 132(1491):78-89. | | |

**Supplemental table 3**. Excess mortality for ethnicity and socioeconomic inequality by overall, sex, enrol age, and clinical measurements

*Excess mortality is presented as per 1,000 person-years. IMD indicates the index of multiple deprivation.*

|  | **All-cause mortality** | **Premature mortality** | **CVD mortality** |
| --- | --- | --- | --- |
|  | **Excess mortality for ethnicity (NZE as reference)** | | |
| **Overall** | -0.83 (-2.50 to 0.85) | 0.63 (-0.62 to 1.88) | -0.26 (-1.12 to 0.60) |
| **Gender** |  |  |  |
| **Men** | -0.83 (-2.47 to 0.82) | 0.74 (-0.65 to 2.13) | -0.26 (-1.12 to 0.59) |
| **Women** | -0.86 (-2.57 to 0.86) | 0.60 (-0.51 to 1.71) | -0.26 (-1.13 to 0.60) |
| **Age stratification** |  |  |  |
| **≤35 years** | -0.21 (-0.77 to 0.34) | 0.45 (-0.39 to 1.28) | -0.06 (-0.29 to 0.18) |
| **>35 years** | -1.11 (-4.00 to 1.77) | 0.92 (-0.79 to 2.63) | -0.37 (-1.90 to 1.16) |
| **Smoking status** |  |  |  |
| **Non-/ex-smoker** | -0.80 (-2.50 to 0.82) | 0.49 (-0.66 to 1.64) | -0.26 (-1.12 to 0.60) |
| **Current smoker** | -0.89 (-2.70 to 0.91) | 0.79 (-1.07 to 2.66) | -0.26 (-1.14 to 0.62) |
| **Enrolment period** |  |  |  |
| **≤2003** | -0.96 (-2.88 to 0.95) | 0.68 (-0.68 to 2.03) | -0.34 (-1.42 to 0.75) |
| **>2003** | -0.56 (-1.69 to 0.56) | 0.51 (-0.52 to 1.53) | -0.11 (-0.45 to 0.24) |
| **Obesity status** |  |  |  |
| **<25 kg/m^2^** | -0.82 (-2.09 to 0.42) | 0.51 (-0.65 to 1.80) | -0.24 (-0.81 to 0.33) |
| **≥25kg/m^2^** | -1.25 (-3.13 to 0.64) | 0.82 (-0.66 to 1.89) | -0.42 (-1.42 to 0.58) |
| **HbA1c** |  |  |  |
| **<70 mmol/mol** | -0.60 (-2.18 to 0.37) | 0.17 (-0.55 to 0.89) | -0.27 (-0.93 to 0.39) |
| **≥70 mmol/mol** | -1.43 (-3.47 to 0.61) | 1.11 (-0.59 to 2.81) | -0.11 (-1.15 to 0.93) |
| **Systolic blood pressure level** |  |  |  |
| **< 120 mmHg** | 0.17 (-0.50 to 0.84) | 0.45 (-0.35 to 1.25) | -0.08 (-0.43 to 0.28) |
| **≥120 mmHg** | -1.08 (-3.49 to 1.33) | 0.90 (-0.69 to 2.49) | -0.27 (-1.49 to 0.95) |
| **Total cholesterol level** |  |  |  |
| **<5.0 mmol/L** | -0.43 (-1.51 to 0.65) | 0.44 (-0.33 to 1.20) | -0.14 (-0.69 to 0.41) |
| **≥5.0 mmol/L** | -1.05 (-3.69 to 1.58) | 1.17 (-0.88 to 3.22) | -0.35 (-1.70 to 1.10) |
| **estimated glomerular filtration rate level** |  |  |  |
| **< 90 ml/min/1.73 m^2^** | 0.59 (-0.33 to 1.51) | 0.61 (-0.37 to 1.58) | 0.31 (-0.04 to 0.66) |
| **≥ 90 ml/min/1.73 m^2^** | -1.39 (-4.22 to 1.44) | 1.05 (-0.65 to 2.74) | -0.58 (-2.22 to 1.06) |
| **Duration of having diabetes** |  |  |  |
| **< 8 years** | -0.39 (-1.40 to 0.63) | 0.58 (-0.52 to 1.68) | -0.12 (-0.63 to 0.38) |
| **≥ 8 years** | -0.87 (-3.11 to 1.38) | 0.72 (-0.66 to 2.11) | -0.29 (-1.45 to 0.88) |
|  | **Excess mortality for socioeconomic (NZDep score quintile 1 as reference)** | | |
| **Overall** |  |  |  |
| Quintile-2 | 1.77 (-0.27 to 3.81) | 1.35 (-0.06 to 2.76) | 0.56 (-0.44 to 1.55) |
| Quintile-3 | 2.94 (0.71 to 3.81) | 1.48 (0.004 to 2.95) | 0.82 (-0.27 to 1.90) |
| Quintile-4 | 3.77 (1.56 to 5.97) | 1.88 (0.42 to 3.34) | 1.17 (0.07 to 2.28) |
| Quintile-5 | 6.94 (4.38 to 9.49) | 4.48 (2.63 to 6.33) | 2.36 (1.01 to 3.71) |
| **Gender: Men** |  |  |  |
| Quintile-2 | 1.77 (-0.27 to 3.82) | 1.47 (-0.09 to 3.03) | 0.57 (-0.45 to 1.58) |
| Quintile-3 | 2.95 (0.69 to 5.21) | 1.63 (-0.01 to 3.27) | 0.83 (-0.29 to 1.95) |
| Quintile-4 | 3.77 (1.52 to 6.02) | 2.09 (0.44 to 3.73) | 1.20 (0.04 to 2.35) |
| Quintile-5 | 6.94 (4.25 to 9.64) | 5.02 (2.81 to 7.22) | 2.42 (0.93 to 3.90) |
| **Gender: Women** |  |  |  |
| Quintile-2 | 1.77 (-0.28 to 3.82) | 1.16 (-0.09 to 2.41) | 0.54 (-0.44 to 1.52) |
| Quintile-3 | 2.94 (0.68 to 5.20) | 1.29 (-0.02 to 2.60) | 0.80 (-0.28 to 1.88) |
| Quintile-4 | 3.76 (1.51 to 6.01) | 1.65 (0.33 to 2.96) | 1.14 (0.03 to 2.26) |
| Quintile-5 | 6.93 (4.26 to 9.60) | 3.96 (2.17 to 5.75) | 2.31 (0.89 to 3.74) |
| **Enrol age: ≤35 years** |  |  |  |
| Quintile-2 | 0.65 (-0.06 to 1.36) | 0.93 (-0.04 to 1.91) | 0.17 (-0.13 to 0.46) |
| Quintile-3 | 0.86 (0.11 to 1.61) | 0.93 (-0.06 to 1.93) | 0.20 (-0.11 to 0.51) |
| Quintile-4 | 1.20 (0.41 to 1.99) | 1.23 (0.22 to 2.24) | 0.31 (-0.04 to 0.66) |
| Quintile-5 | 2.24 (1.21 to 3.26) | 2.95 (1.54 to 4.37) | 0.63 (0.11 to 1.14) |
| **Enrol age: >35 years** |  |  |  |
| Quintile-2 | 3.39 (-0.21 to 6.99) | 1.90 (-0.05 to 3.86) | 1.09 (-0.74 to 2.92) |
| Quintile-3 | 4.46 (0.75 to 8.17) | 1.90 (-0.08 to 3.87) | 1.29 (-0.57 to 3.15) |
| Quintile-4 | 6.24 (2.47 to 10.02) | 2.51 (0.51 to 4.50) | 2.02 (0.06 to 3.97) |
| Quintile-5 | 11.62 (7.24 to 16.01) | 6.02 (3.42 to 8.61) | 4.11 (1.71 to 6.52) |
| **Smoking status: non-/ex-smoker** |  |  |  |
| Quintile-2 | 1.78 (-0.27 to 3.82) | 1.29 (-0.06 to 2.63) | 0.56 (-0.44 to 1.57) |
| Quintile-3 | 2.97 (0.72 to 5.22) | 1.36 (-0.04 to 2.76) | 0.83 (-0.27 to 1.94) |
| Quintile-4 | 3.79 (1.56 to 6.02) | 1.74 (0.35 to 3.13) | 1.19 (0.07 to 2.32) |
| Quintile-5 | 7.00 (4.38 to 9.63) | 4.07 (2.28 to 5.86) | 2.43 (1.01 to 3.84) |
| **Smoking status: Current smoker** |  |  |  |
| Quintile-2 | 1.70 (-0.33 to 3.73) | 1.90 (-0.21 to 4.02) | 0.49 (-0.44 to 1.42) |
| Quintile-3 | 2.84 (0.54 to 5.14) | 2.02 (-0.15 to 4.19) | 0.73 (-0.33 to 1.79) |
| Quintile-4 | 3.63 (1.25 to 6.00) | 2.58 (0.36 to 4.80) | 1.04 (-0.12 to 2.21) |
| Quintile-5 | 6.70 (3.62 to 9.78) | 6.03 (2.88 to 9.19) | 2.12 (0.41 to 3.83) |
| **Enrolment period: ≤2003** |  |  |  |
| Quintile-2 | 1.79 (-0.54 to 4.13) | 1.44 (-0.09 to 2.97) | 0.81 (-0.48 to 2.09) |
| Quintile-3 | 3.13 (0.59 to 5.67) | 1.54 (-0.05 to 3.13) | 0.91 (-0.42 to 2.24) |
| Quintile-4 | 4.29 (1.72 to 6.86) | 2.03 (0.42 to 3.63) | 1.45 (0.05 to 2.85) |
| Quintile-5 | 7.97 (4.97 to 10.97) | 4.85 (2.78 to 6.92) | 2.96 (1.23 to 4.69) |
| **Enrolment period: >2003** |  |  |  |
| Quintile-2 | 1.03 (-0.34 to 2.41) | 1.08 (-0.11 to 2.27) | 0.28 (-0.20 to 0.76) |
| Quintile-3 | 1.81 (0.26 to 3.35) | 1.16 (-0.10 to 2.41) | 0.32 (-0.19 to 0.83) |
| Quintile-4 | 2.47 (0.88 to 4.06) | 1.52 (0.24 to 2.80) | 0.51 (-0.06 to 1.08) |
| Quintile-5 | 4.60 (2.58 to 6.62) | 3.65 (1.80 to 5.49) | 1.04 (0.19 to 1.89) |
| **Obesity status: <25 kg/m^2^** |  |  |  |
| Quintile-2 | 1.40 (-0.25 to 3.04) | 1.31 (-0.09 to 2.72) | 0.40 (-0.34 to 1.13) |
| Quintile-3 | 2.24 (0.41 to 4.06) | 1.43 (-0.05 to 2.92) | 0.56 (-0.25 to 1.36) |
| Quintile-4 | 2.97 (1.12 to 4.82) | 1.83 (0.34 to 3.32) | 0.83 (-0.03 to 1.69) |
| Quintile-5 | 5.35 (3.06 to 7.64) | 4.35 (2.24 to 6.46) | 1.64 (0.46 to 2.82) |
| **Obesity status: ≥25 kg/m^2^** |  |  |  |
| Quintile-2 | 1.97 (-0.32 to 4.27) | 1.37 (-0.07 to 2.81) | 0.65 (-0.52 to 1.81) |
| Quintile-3 | 3.16 (0.67 to 5.65) | 1.49 (-0.01 to 2.99) | 0.90 (-0.35 to 2.16) |
| Quintile-4 | 4.20 (1.70 to 6.70) | 1.90 (0.40 to 3.41) | 1.36 (0.05 to 2.66) |
| Quintile-5 | 7.56 (4.69 to 10.43) | 4.53 (2.60 to 6.46) | 2.67 (1.09 to 4.25) |
| **HbA1c level: <70 mmol/mol** |  |  |  |
| Quintile-2 | 1.45 (-0.25 to 3.15) | 0.86 (-0.08 to 1.79) | 0.47 (-0.39 to 1.33) |
| Quintile-3 | 2.42 (0.54 to 4.30) | 0.94 (-0.04 to 1.91) | 0.69 (-0.25 to 1.63) |
| Quintile-4 | 2.90 (1.03 to 4.77) | 1.05 (0.10 to 2.01) | 0.94 (-0.03 to 1.92) |
| Quintile-5 | 5.32 (3.06 to 7.59) | 2.52 (1.18 to 3.85) | 1.90 (0.62 to 3.18) |
| **HbA1c level: ≥70 mmol/mol** |  |  |  |
| Quintile-2 | 2.11 (-0.36 to 4.57) | 1.90 (-0.13 to 3.92) | 0.65 (-0.53 to 1.83) |
| Quintile-3 | 3.51 (0.80 to 6.23) | 2.07 (-0.05 to 4.19) | 0.95 (-0.34 to 2.25) |
| Quintile-4 | 4.21 (1.60 to 6.83) | 2.33 (0.33 to 4.33) | 1.31 (0.02 to 2.60) |
| Quintile-5 | 7.73 (4.72 to 10.73) | 5.57 (3.09 to 8.04) | 2.63 (1.05 to 4.20) |
| **Systolic blood pressure level: <120mmHg** |  |  |  |
| Quintile-2 | 0.72 (-0.13 to 1.57) | 0.87 (-0.07 to 1.82) | 0.23 (-0.20 to 0.67) |
| Quintile-3 | 0.98 (0.07 to 1.89) | 0.86 (-0.10 to 1.83) | 0.29 (-0.17 to 0.75) |
| Quintile-4 | 1.47 (0.50 to 2.15) | 1.19 (0.19 to 2.20) | 0.48 (-0.04 to 1.00) |
| Quintile-5 | 2.68 (1.42 to 3.93) | 2.83 (1.39 to 4.27) | 0.95 (0.21 to 1.69) |
| **Systolic blood pressure level: ≥120mmHg** |  |  |  |
| Quintile-2 | 2.55 (-0.41 to 5.51) | 1.70 (-0.09 to 3.50) | 0.80 (-0.64 to 2.23) |
| Quintile-3 | 3.49 (0.42 to 6.56) | 1.68 (-0.12 to 3.49) | 0.99 (-0.49 to 2.47) |
| Quintile-4 | 5.25 (2.08 to 8.43) | 2.33 (0.48 to 4.18) | 1.63 (0.05 to 3.22) |
| Quintile-5 | 9.55 (5.90 to 13.19) | 5.52 (3.15 to 7.88) | 3.26 (1.34 to 5.18) |
| **Total cholesterol level: <5.0 mmol/L** |  |  |  |
| Quintile-2 | 1.23 (-0.13 to 2.58) | 0.88 (-0.03 to 1.78) | 0.38 (-0.28 to 1.04) |
| Quintile-3 | 1.83 (0.36 to 3.29) | 0.87 (-0.05 to 1.80) | 0.51 (-0.20 to 1.22) |
| Quintile-4 | 2.40 (0.92 to 3.87) | 1.14 (0.20 to 2.07) | 0.74 (-0.0002 to 1.49) |
| Quintile-5 | 4.28 (2.52 to 6.04) | 2.64 (1.37 to 3.91) | 1.46 (0.50 to 2.42) |
| **Total cholesterol level: ≥5.0 mmol/L** |  |  |  |
| Quintile-2 | 2.95 (-0.30 to 6.19) | 2.29 (-0.05 to 4.62) | 0.92 (-0.68 to 2.51) |
| Quintile-3 | 4.38 (0.92 to 7.84) | 2.27 (-0.08 to 4.63) | 1.23 (-0.47 to 2.92) |
| Quintile-4 | 5.74 (2.28 to 9.21) | 2.96 (0.59 to 5.33) | 1.80 (0.05 to 3.55) |
| Quintile-5 | 10.27 (6.28 to 14.25) | 6.86 (3.86 to 9.87) | 3.54 (1.40 to 5.68) |
| **estimated glomerular filtration rate level: < 90 ml/min/1.73 m^2^** |  |  |  |
| Quintile-2 | 0.92 (-0.13 to 1.97) | 1.02 (-0.05 to 2.10) | 0.21 (-0.17 to 0.58) |
| Quintile-3 | 1.50 (0.33 to 2.67) | 1.11 (-0.02 to 2.24) | 0.30 (-0.12 to 0.72) |
| Quintile-4 | 1.99 (0.80 to 3.18) | 1.43 (0.30 to 2.57) | 0.45 (-0.01 to 0.90) |
| Quintile-5 | 3.86 (2.35 to 5.36) | 3.50 (1.95 to 5.05) | 0.96 (0.30 to 1.61) |
| **estimated glomerular filtration rate level: ≥ 90 ml/min/1.73 m^2^** |  |  |  |
| Quintile-2 | 2.94 (-0.37 to 6.25) | 1.79 (-0.08 to 3.66) | 1.03 (-0.77 to 2.82) |
| Quintile-3 | 4.77 (1.15 to 8.39) | 1.94 (-0.01 to 3.89) | 1.47 (-0.49 to 3.42) |
| Quintile-4 | 6.33 (2.68 to 9.98) | 2.51 (0.54 to 4.47) | 2.19 (0.16 to 4.23) |
| Quintile-5 | 12.27 (7.81 to 16.73) | 6.11 (3.43 to 8.80) | 4.71 (2.04 to 7.37) |
| **Duration of having diabetes: < 8 years** |  |  |  |
| Quintile-2 | 1.07 (-0.17 to 2.30) | 1.20 (-0.07 to 2.47) | 0.33 (-0.27 to 0.92) |
| Quintile-3 | 1.70 (0.34 to 3.07) | 1.30 (-0.03 to 2.63) | 0.46 (-0.19 to 1.11) |
| Quintile-4 | 2.32 (0.91 to 3.72) | 1.67 (0.34 to 3.01) | 0.70 (-0.002 to 1.40) |
| Quintile-5 | 4.09 (2.36 to 5.81) | 3.95 (2.11 to 5.79) | 1.35 (0.41 to 2.30) |
| **Duration of having diabetes: ≥ 8 years** |  |  |  |
| Quintile-2 | 2.37 (-0.35 to 5.09) | 1.48 (-0.08 to 3.03) | 0.75 (-0.59 to 2.10) |
| Quintile-3 | 3.79 (0.84 to 6.74) | 1.60 (-0.02 to 3.22) | 1.06 (-0.39 to 2.52) |
| Quintile-4 | 5.15 (2.16 to 8.15) | 2.07 (0.43 to 3.70) | 1.61 (0.09 to 3.14) |
| Quintile-5 | 9.09 (5.66 to 12.52) | 4.88 (2.76 to 7.00) | 3.14 (1.29 to 4.98) |

**Supplemental table 4**. Adjusted Mortality rates ration and excess mortality for ethnicity and socioeconomic inequality

*Excess mortality is presented as per 1,000 person-years. IMD indicates the index of multiple deprivation.*

*Age, sex, enrolment period, obesity status, blood pressure, smoking status, duration of having diabetes, HbA1c, total cholesterol, and estimated glomerular filtration rate levels were adjusted.*

|  | **All-cause mortality** | **Premature mortality** | **CVD mortality** | |
| --- | --- | --- | --- | --- |
|  | **Adjusted mortality rates ratio for ethnicity (NZE as reference)** | | | |
| **Non-NZE** | 0.93 (0.71 to 1.22) | 0.73 (0.50 to 1.05) | 0.99 (0.58 to1.68) | |
|  | **Adjusted excess mortality for ethnicity (NZE as reference)** | | | |
| **Non-NZE** | -0.95 (-4.62 to 2.72) | -1.71 (-3.83 to 0.41) | | -0.04 (-1.93 to 1.86) |
|  | **Adjusted excess mortality for socioeconomic (NZDep score quintile-1 as reference)** | | | |
| Quintile-2 | 5.41 (0.89 to 9.93) | 2.41 (0.02 to 4.80) | 1.64 (--0.62 to 3.89) | |
| Quintile-3 | 5.24 (0.93 to 9.55) | 2.54 (0.07 to 5.01) | 1.51 (-0.61 to 3.63) | |
| Quintile-4 | 7.39 (3.01 to 11.77) | 2.93 (0.58 to 5.28) | 2.34 (0.12 to 4.56) | |
| Quintile-5 | 12.23 (7.43 to 17.02) | 6.77 (3.87 to 9.67) | 4.33 (1.77 to 6.90) | |
|  | **Adjusted excess mortality for ethnicity (NZDep score quintile-1 as reference)** | | | |
| Quintile-2 | 1.75 (1.11-2.76) | 2.01 (1.02-3.96) | 2.04 (0.79-5.26) | |
| Quintile-3 | 1.73 (1.11-2.70) | 2.06 (1.04-4.09) | 1.96 (0.77-4.98) | |
| Quintile-4 | 2.03 (1.33-3.10) | 2.22 (1.15-4.29) | 2.48 (1.03-5.99) | |
| Quintile-5 | 2.70 (1.79-4.07) | 3.83 (2.05-7.13) | 3.75 (1.61-8.72) | |

**Supplemental Table 5**  Population attributable fractions of mortality restriction by socioeconomic deprivation and ethnicity

|  | **All-cause mortality** | **Premature mortality** | **CVD mortality** |
| --- | --- | --- | --- |
| **Ethnicity** |  |  |  |
| No adjustment | -4.33 (-12.39 to 3.15) % | 5.36 (-6.90 to 16.22) % | -5.02 (-20.97 to 8.83) % |
| Adjustment |  |  |  |
| Socioeconomic group | -3.08 (-11.45 to 4.66) % | 5.64 (-7.00 to 16.79) % | -5.70 (-22.71 to 8.96) % |
| Socioeconomic group, age, sex | 3.87 (-3.78 to 10.96) % | 8.79 (-3.47 to 19.59) % | -2.43 (-13.05 to 15.78) % |
| Socioeconomic group, age, sex, duration of having diabetes, enrolment year, smoking status | 4.00 (-3.64 to 11.09) % | 8.27 (-4.12 to 19.19) % | 3.22 (-12.00 to 16.37) % |
| Socioeconomic group, age, sex, duration of having diabetes, enrolment year, smoking status, BMI, SBP, DBP | 5.03 (-3.52 to 12.87) % | 10.82 (-2.69 to 22.55) % | -0.23 (-17.06 to 14.18) % |
| Socioeconomic group, age, sex, duration of having diabetes, enrolment year, smoking status, BMI, SBP, DBP, HbA1c | 2.15 (-7.43 to 10.88) % | 5.08 (-10.60 to 18.54) % | 2.66 (-16.00 to 18.32) % |
| Socioeconomic group, age, sex, duration of having diabetes, enrolment year, smoking status, BMI, SBP, DBP, HbA1c, TC, triglyceride, LDL-C, HDL-C | 4.85 (-8.29 to 16.40) % | 9.36 (-13.58 to 27.66) % | -4.17 (-34.08 to 19.06) % |
| Socioeconomic group, age, sex, duration of having diabetes, enrolment year, smoking status, BMI, SBP, DBP, HbA1c, TC, triglyceride, LDL-C, HDL-C, eGFR | 5.22 (-8.09 to 16.90) % | 9.01 (-14.02 to 27.39) % | -5.72 (-36.51 to 18.12) % |
| Socioeconomic group, age, sex, duration of having diabetes, enrolment year, smoking status, BMI, SBP, DBP, HbA1c, TC, triglyceride, LDL-C, HDL-C, eGFR, statin and antihypertensive medicine | 5.93 (-8.47 to 18.43) % | 12.03 (-11.64 to 30.68) % | -13.02 (-57.74 to 19.03) % |
| **Socioeconomic deprivation** |  |  |  |
| No adjustment | 46.60 (26.11 to 61.41) % | 55.99 (26.39 to 73.68) % | 55.82 (10.70 to 78.14) % |
| Adjustment |  |  |  |
| Ethnicity | 47.16 (26.61 to 61.96) % | 55.11 (24.54 to 73.30) % | 56.64 (11.24 to 78.82) % |
| Ethnicity, age, sex | 41.86 (19.42 to 58.05) % | 54.20 (23.07 to 72.74) % | 51.79 (2.60 to 76.14) % |
| Ethnicity, age, sex, duration of having diabetes, enrolment year, smoking status | 41.27 (18.61 to 57.62) % | 53.37 (21.54 to 72.28) % | 51.69 (2.14 to 76.15) % |
| Ethnicity, age, sex, duration of having diabetes, enrolment year, smoking status, BMI, SBP, DBP | 31.90 (2.01 to 52.67) % | 36.04 (3.18 to 57.75) % | 50.44 (4.62 to 74.25) % |
| Ethnicity, age, sex, duration of having diabetes, enrolment year, smoking status, BMI, SBP, DBP, HbA1c | 28.20 (2.42 to 47.17) % | 28.62 (2.46 to 47.76) % | 52.92 (3.50 to 77.03) % |
| Ethnicity, age, sex, duration of having diabetes, enrolment year, smoking status, BMI, SBP, DBP, HbA1c, TC, triglyceride, LDL-C, HDL-C | 26.21 (1.04 to 44.98) % | 25.50 (0.18 to 44.40) % | 47.29 (3.94 to 71.07) % |
| Ethnicity, age, sex, duration of having diabetes, enrolment year, smoking status, BMI, SBP, DBP, HbA1c, TC, triglyceride, LDL-C, HDL-C, eGFR | 24.61 (0.95 to 42.61) % | 24.72 (1.09 to 42.71) % | 52.06 (3.60 to 76.16) % |
| Ethnicity, age, sex, duration of having diabetes, enrolment year, smoking status, BMI, SBP, DBP, HbA1c, TC, triglyceride, LDL-C, HDL-C, eGFR, statin and antihypertensive medicine | 25.74 (0.84 to 44.39) % | 25.88 (0.69 to 44.69) % | 55.89 (1.20 to 80.31) % |

**Supplemental Table 6.** Mortality rates ratio (MRR) of clinical events among people with type 1 diabetes in DCSS between 1994-2018

MRR was standardized against DCSS type 2 diabetes population. N/A indicates the no recorded events for the sub-group people with type 1 diabetes. MRR was presented as MRR (95% confidence interval).

|  | **All-cause mortality** | | | **Premature mortality** | | | **CVD mortality** | | |
| --- | --- | --- | --- | --- | --- | --- | --- | --- | --- |
|  | **ALL** | **NZE** | **Non-NZE** | **ALL** | **NZE** | **Non-NZE** | **ALL** | **NZE** | **Non-NZE** |
| **Overall** |  |  |  |  |  |  |  |  |  |
| **All** | 0.39 (0.34 to 0.45) | 0.32 (0.28 to 0.38) | 0.42 (0.32 to 0.55) | 0.65 (0.52 to 0.80) | 1.02 (0.76 to 1.37) | 0.59 (0.41 to 0.84) | 0.31 (0.24 to 0.41) | 0.24 (0.18 to 0.32) | 0.36 (0.21 to 0.60) |
| **Male** | 0.38 (0.31 to 0.45) | 0.32 (0.26 to 0.39) | 0.43 (0.30 to 0.62) | 0.69 (0.52 to 0.91) | 1.02 (0.69 to 1.51) | 0.70 (0.44 to 1.11) | 0.31 (0.22 to 0.45) | 0.22 (0.15 to 0.34) | 0.47 (0.25 to 0.88) |
| **Female** | 0.41 (0.33 to 0.50) | 0.34 (0.26 to 0.43) | 0.41 (0.28 to 0.61) | 0.60 (0.43 to 0.83) | 1.03 (0.65 to 1.62) | 0.45 (0.25 to 0.79) | 0.31 (0.21 to 0.46) | 0.26 (0.17 to 0.40) | 0.23 (0.09 to 0.56) |
|  |  |  |  |  |  |  |  |  |  |
| **<25** |  |  |  |  |  |  |  |  |  |
| **All** | 0.44 (0.26 to 0.75) | 0.50 (0.24 to 1.03) | 0.52 (0.22 to 1.23) | 0.43 (0.25 to 0.74) | 0.50 (0.24 to 1.03) | 0.52 (0.22 to 1.23) | 0.33 (0.11 to 1.03) | N/A | 0.59 (0.14 to 2.45) |
| **Male** | 0.34 (0.15 to 0.76) | N/A | 0.43 (0.13 to 1.39) | 0.33 (0.15 to 0.74) | N/A | 0.43 (0.13 to 1.39) | 0.25 (0.08 to 0.75) | N/A | 0.48 (0.12 to 1.92) |
| **Female** | 0.53 (0.25 to 1.10) | 0.33 (0.14 to 0.77) | 0.59 (0.17 to 2.04) | 0.52 (0.25 to 1.09) | 0.33 (0.14 to 0.77) | 0.59 (0.17 to 2.04) | N/A | N/A | N/A |
|  |  |  |  |  |  |  |  |  |  |
| **25-34** |  |  |  |  |  |  |  |  |  |
| **All** | 0.78 (0.47 to 1.29) | 0.49 (0.28 to 0.85) | 1.06 (0.42 to 2.72) | 0.79 (0.48 to 1.32) | 0.49 (0.28 to 0.85) | 1.06 (0.42 to 2.72) | 0.60 (0.22 to 1.65) | 0.50 (0.17 to 1.45) | 0.42 (0.04 to 4.37) |
| **Male** | 0.61 (0.32 to 1.17) | 0.92 (0.40 to 2.08) | 0.55 (0.16 to 1.88) | 0.63 (0.33 to 1.19) | 0.92 (0.40 to 2.08) | 0.55 (0.16 to 1.88) | 0.71 (0.23 to 2.25) | 0.63 (0.20 to 1.91) | N/A |
| **Female** | 0.94 (0.43 to 2.06) | 0.31 (0.13 to 0.73) | 1.94 (0.43 to 8.73) | 0.92 (0.42 to 2.02) | 0.31 (0.13 to 0.73) | 1.94 (0.43 to 8.73) | 0.25 (0.03 to 2.24) | N/A | 1.00 (0.06 to 15.99) |
|  |  |  |  |  |  |  |  |  |  |
| **35-44** |  |  |  |  |  |  |  |  |  |
| **All** | 0.66 (0.45 to 0.97) | 0.83 (0.51 to 1.34) | 0.64 (0.31 to 1.31) | 0.65 (0.44 to 0.96) | 0.80 (0.49 to 1.30) | 0.65 (0.32 to 1.35) | 0.43 (0.20 to 0.94) | 0.38 (0.14 to 1.06) | 0.67 (0.19 to 2.38) |
| **Male** | 0.57 (0.35 to 0.94) | 0.76 (0.40 to 1.43) | 0.72 (0.29 to 1.79) | 0.58 (0.36 to 0.95) | 0.76 (0.40 to 1.43) | 0.74 (0.30 to 1.85) | 0.44 (0.18 to 1.06) | 0.28 (0.08 to 1.03) | 1.01 (0.25 to 4.06) |
| **Female** | 0.77 (0.41 to 1.44) | 0.96 (0.45 to 2.05) | 0.50 (0.15 to 1.66) | 0.73 (0.38 to 1.38) | 0.88 (0.41 to 1.92) | 0.52 (0.15 to 1.73) | 0.33 (0.07 to 1.65) | 0.59 (0.10 to 1.38) | N/A |
|  |  |  |  |  |  |  |  |  |  |
| **45-54** |  |  |  |  |  |  |  |  |  |
| **All** | 0.76 (0.54 to 1.09) | 0.91 (0.59 to 1.41) | 0.69 (0.37 to 1.31) | 0.79 (0.52 to 1.21) | 1.02 (0.58 to 1.78) | 0.80 (0.39 to 1.66) | 0.85 (0.45 to 1.62) | 1.06 (0.47 to2.39) | 0.74 (0.23 to 2.36) |
| **Male** | 0.83 (0.52 to 1.31) | 0.88 (0.49 to 1.55) | 0.86 (0.38 to 1.92) | 1.00 (0.58 to 1.72) | 1.13 (0.55 to 2.30) | 1.09 (0.45 to 2.67) | 0.58 (0.23 to 1.48) | 0.56 (0.17 to 1.92) | 0.78 (0.17 to 3.55) |
| **Female** | 0.68 (0.39 to 1.19) | 0.97 (0.49 to 1.93) | 0.50 (0.17 to 1.45) | 0.57 (0.28 to 1.16) | 0.87 (0.36 to 2.14) | 0.43 (0.11 to 1.65) | 1.11 (0.45 to 2.73) | 1.94 (0.59 to 1.37) | 0.69 (0.11 to 4.22) |
|  |  |  |  |  |  |  |  |  |  |
| **55+** |  |  |  |  |  |  |  |  |  |
| **All** | 0.81 (0.64 to 1.02) | 0.81 (0.62 to 1.05) | 0.70 (0.44 to 1.09) | 1.00 (0.43 to 2.31) | 2.07 (0.51 to 8.43) | 0.84 (0.26 to 2.77) | 0.64 (0.42 to 0.96) | 0.63 (0.40 to 1.00) | 0.50 (0.20 to 1.23) |
| **Male** | 0.72 (0.53 to 0.98) | 0.75 (0.53 to 1.06) | 0.54 (0.28 to 1.03) | 1.40 (0.44 to 4.41) | 1.80 (0.27 to 11.91) | 1.41 (0.31 to 6.49) | 0.55 (0.30 to 0.99) | 0.53 (0.27 to 1.03) | 0.51 (0.15 to 1.69) |
| **Female** | 0.93 (0.66 to 1.30) | 0.89 (0.60 to 1.31) | 0.90 (0.48 to 1.70) | 0.80 (0.21 to 2.98) | 2.44 (0.30 to 19.93) | 0.34 (0.04 to 3.27) | 0.77 (0.43 to 1.38) | 0.76 (0.40 to 1.43) | 0.48 (0.12 to 1.91) |
